# Fractionated Lignosulfonates for Laccase-Catalyzed Oxygen-Scavenging Films and Coatings

**DOI:** 10.3390/molecules26206322

**Published:** 2021-10-19

**Authors:** Sandra Winestrand, Lars Järnström, Leif J. Jönsson

**Affiliations:** 1 Department of Chemistry, Umeå University, SE-901 87 Umeå, Sweden; sandra.winestrand@umu.se; 2 Department of Chemical Engineering, Karlstad University, SE-651 88 Karlstad, Sweden; lars.jarnstrom@kau.se

**Keywords:** coating, paperboard, active packaging, lignin, lignosulfonates, laccase, oxygen scavenging

## Abstract

Lignin derivatives have potential as antioxidants in advanced packaging materials through their ability to scavenge oxygen in reactions catalyzed by phenol-oxidizing enzymes, such as laccase. The effects of size fractionation of lignosulfonates on laccase-catalyzed reactions were investigated in experiments with aqueous solutions, films, and coated paperboard. Four industrial lignosulfonate preparations were compared: *Feed* (unfractionated), *Prod* (5–60 kDa enriched), *Conc* (≥60 kDa enriched), and *Perm* (≤60 kDa enriched). Extraction of lignosulfonates from films showed that the enzymic reaction increased the average molecular weight from <10,000 to up to 66,000. The enzymatic reaction resulted in an increase in the water contact angle of the films from the range 25–49° to 56–81°. The four preparations showed relatively small differences with regard to their ability to scavenge oxygen in aqueous solution and in experiments with coated paperboards in sealed chambers. Coatings with lignosulfonates where the contents of low-molecular weight material had been reduced (i.e., *Prod* and *Conc*) showed improved water resistance after the enzymic reaction. Thus, in both aqueous and solid media, fractionation of lignosulfonates had little effect on oxygen scavenging, but fractionation was beneficial for other reasons, such as improved cross-linking resulting in higher molecular weight and superior water resistance.

## 1. Introduction

Lignin is one of the main constituents of wood and is a major residue from chemical pulping processes [1,2,3,4,5]. Although useful as an energy source, it would be highly advantageous to utilize at least a part of the lignin for advanced high value-added products. Lignosulfonates are co-products of sulfite pulping and are mainly used as additives to concrete [3,4,6]. While many other lignin derivates show poor solubility in water, the sulfonation that occurs during sulfite pulping renders lignosulfonates soluble even under neutral and moderately acidic conditions. Previous studies indicate that lignosulfonates and other lignin derivatives have potential as antioxidants in enzyme-catalyzed oxygen-scavenging formulations, for example, in advanced packaging systems [7]. Lignin fractions may also be used as antioxidants due to their free radical and oxygen radical scavenging properties [8].

Laccase is a phenol-oxidizing enzyme that catalyzes the oxidation of phenolic lignin derivatives, such as lignosulfonates, and reduces molecular oxygen to water [9,10,11,12,13]. Thus, addition of laccase to lignin derivatives makes it possible to utilize them as antioxidants in a more efficient way; without a functional catalyst, the reaction is too slow to be useful. There are many other enzymes that catalyze scavenging of oxygen and that have been studied with regard to active packaging, such as glucose oxidase [14] and oxalate oxidase [15]. Laccase has, however, some distinct advantages [16], which include the possibility of using lignin derivatives as substrates and reduction of molecular oxygen to water rather than to hydrogen peroxide. With regard to enzymes for active packaging, where the aim is to prolong the shelf-life of packaged food inside an oxygen barrier, most studies have been made with glucose oxidase [14,17,18,19,20], which oxidizes glucose to gluconolactone. However, as glucose oxidase reduces oxygen to toxic hydrogen peroxide, this may necessitate incorporation of a second catalytic system, such as catalase, which scavenges hydrogen peroxide. While scavenging of hydrogen peroxide is in no way impossible to achieve, the reaction catalyzed by laccase is more straightforward. Furthermore, laccase-catalyzed oxidation of lignin derivatives causes creation of radicals, which can couple with each other, leading to cross-linking and formation of larger molecules, in a manner reminiscent of oxidative coupling reactions in lignin biosynthesis [9,12]. In this way, a coating or a film that contains lignin derivatives and laccase will acquire new properties through improved cross-linking [16].

Previous research on laccase and oxygen scavenging for active packaging has focused on small phenolic compounds [21], and on the possibility to use polymeric substances such as lignosulfonates and other technical lignin preparations including Kraft lignin, organosolv lignin, and hydrolysis lignin [7,16]. However, there are no studies to date on how the properties of a certain type of lignin derivative affect the reaction. The aim of this study was to investigate how fractionation of an industrial lignosulfonate preparation affects laccase-catalyzed reactions in aqueous solution, paperboard coatings, and films. Apart from oxygen scavenging in liquid and solid media, water stability and mechanical properties were also evaluated. The effects on size distribution of the lignosulfonates and the water contact angle, which is a measure of the hydrophobicity, were also studied. An advantage of using authentic industrial lignosulfonate fractions is that it increases the industrial relevance of the results. As reactions catalyzed by laccase are typically carried out at slightly acidic pH, the relatively high solubility of lignosulfonates under such conditions is an advantage. Research in this area can lead to new possibilities to use lignin from wood biorefinery operations, innovations in active packaging, and increased understanding of the importance of the fundamental properties of lignin derivatives that serve as antioxidants in enzyme-catalyzed reactions.

## 2. Results and Discussion

The influence of size fractionation of lignosulfonates on oxygen-scavenging catalyzed by the phenol-oxidizing enzyme laccase was investigated in aqueous solutions, films, and paperboard coatings. The properties of the lignosulfonate preparations (Table 1) were studied with regard to the phenolic content and the average molecular weight. The investigation also covered the effects of the enzymatic reaction on the average molecular weight of the lignosulfonates, the contact angle of lignosulfonate-containing films, the mechanical properties of lignosulfonate-containing films, the capability to scavenge oxygen, and the water resistance of lignosulfonate-containing films.

The estimate of the phenolic content of the lignosulfonate fractions is presented in Table 2. The highest phenolic content was found in the *Prod* fraction, where the content was 0.31%, and the lowest phenolic content was detected in the *Conc* and *Perm* fractions, where the phenolic content was 0.25%. Industrial lignosulfonate preparations may contain not only sulfonated lignin with phenolic groups, but also fragmented carbohydrates, carbohydrate derivatives, and salts that come from the sulfite cooking process.

**Figure 1:**
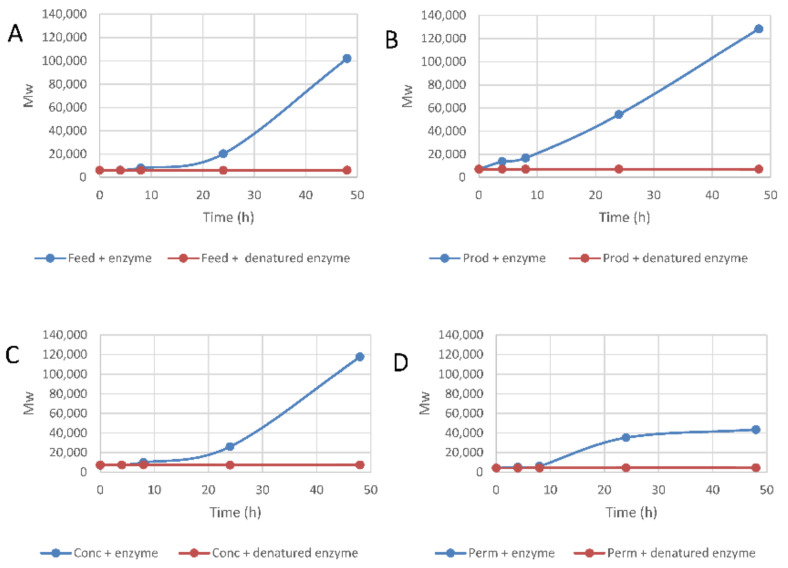
Plots showing M_w_ (weight average molecular weight) values determined using GPC as a function of reaction time. The four lignosulfonate preparations included were (**A**) *Feed*, (**B**) *Prod*, (**C**) *Conc*, and (**D**) *Perm*. Reactions were performed with active (blue symbols) or denatured (red symbols) laccase. M_w_ values represent poly(styrenesulfonate) equivalents determined using UV detection.

The effects of laccase-treatment on the size distribution of lignosulfonate in aqueous solution were analyzed using GPC and the results are shown in Figure 1. The initial average molecular weight was always <10,000 and did not increase in the absence of active laccase. It is noteworthy in this context that industrial ultrafiltration of lignosulfonates is not an exhaustive process, i.e., there will be an enrichment of larger molecules in the retentate and an enrichment of smaller molecules in the permeate, but not an absolute exclusion based on size. Therefore, it is not surprising that the average molecular weight of the *Conc* fraction was far below 60,000. All four lignosulfonate preparations showed higher average molecular weight after laccase-treatment during 24 h and 48 h (Figure 1). For the *Prod* fraction (Figure 1B), there was an obvious increase already after 4 and 8 h. The *Feed* showed an increase from 6000 at 0 h to 102,000 after 48 h reaction time (Figure 1A), while the average molecular weight of the *Prod* fraction increased from 7000 to 128,000 after 48 h reaction time (Figure 1B). The average molecular weight of the *Conc* fraction increased from 7100 to 118,000 after 48 h (Figure 1C). The increase in average molecular weight was lower for the *Perm* fraction, which changed from 4300 to 43,500 after 48 h reaction time (Figure 1D). The lignosulfonate preparations with less low-molecular-weight material (i.e., *Prod* and *Conc*) showed a higher average molecular weight after 48 h reaction time compared to *Feed* and *Perm*. Thus, removal of small-sized lignosulfonates made the effect of cross-linking on molecular weight more evident.

The average molecular weight of lignosulfonate was also analyzed in the cast films and the results are shown in Table 3. The average molecular weight increased in all films containing active laccase compared to the control films containing denatured laccase. Without active catalyst, the average molecular weight was <10,000. As for experiments in liquid medium (Figure 1), the *Perm* fraction had the lowest value. The lignosulfonate preparations where the low-molecular-weight fraction was depleted (*Prod* and *Conc*) showed a higher average molecular weight compared to *Feed* and *Perm*. The highest value was observed for the *Prod* fraction, where the average molecular weight increased to 66,000.

**Figure 2:**
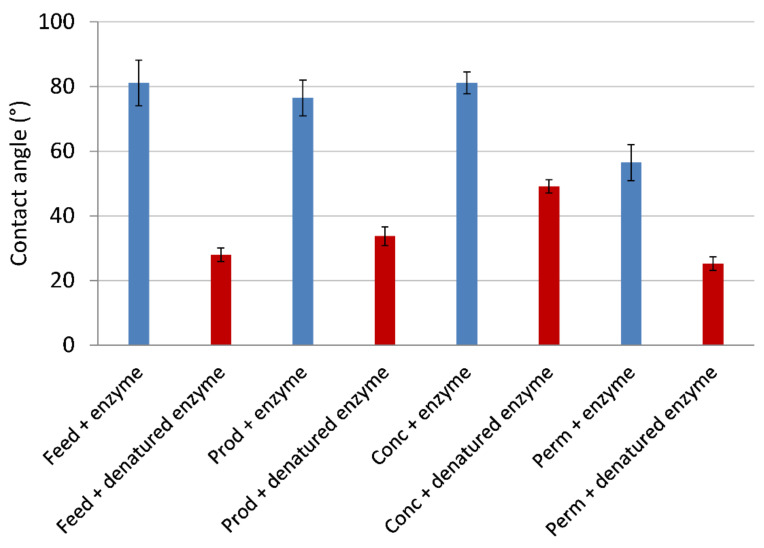
The water contact angle of films with different lignosulfonate preparations (*Feed*, *Prod*, *Conc*, and *Perm*) and active (**blue bars**) or denatured (**red bars**) laccase.

The water contact angle was analyzed using cast films and the results are shown in Figure 2. Without an enzymatic reaction (denatured enzyme), the contact angle for preparations where low-molecular-weight material had not been depleted (i.e., *Perm* and *Feed*) was lower (25–28°) than the contact angle of the two others (34–49°). This indicates that enrichment of high-molecular-weight material was associated with increased hydrophobicity, which, as expected, was most evident for the *Conc* fraction (contact angle 49°). Another reason for the relatively low contact angles of films with *Perm* and *Feed* could tentatively be higher sugar content. Borrega et al. [22] reported that the contact angle decreased with increasing sugar content. However, since the films used in the contact angle experiments (Figure 2) were based on formulations in which starch was the predominant component, potential effects of sugar on contact angle are expected to become obscured by the high starch content. The important feature shown in Figure 2 is the dramatic increase in contact angle due to the enzyme-catalyzed reaction between lignin moieties. For all films containing active enzyme, the contact angle was higher (56–81°) compared to the control containing denatured enzyme (25–49°). The higher contact angle for the enzyme-treated films indicates that the material becomes more hydrophobic after the enzymatic treatment, which agrees with the GPC results, showing increased average molecular weight. It has been reported elsewhere that potato starch films cross-linked by UV radiation possess higher water contact angle after cross-linking than before [23]. This was explained in terms of restriction in segmental mobility. Figure 2 clearly shows similar results resulting in enzyme-catalyzed cross-linking of lignosulfonate-starch free standing films. It is likely that the explanation to the behavior shown in Figure 2 for the lignosulfonate-starch films is restriction in segmental mobility.

The mechanical properties of films with lignosulfonate fractions and with or without active laccase were investigated using DMA (Dynamic Mechanical Analysis). However, no significant difference in the storage modulus (E’) between films containing active and denatured enzyme was detected (data not shown).

**Figure 3:**
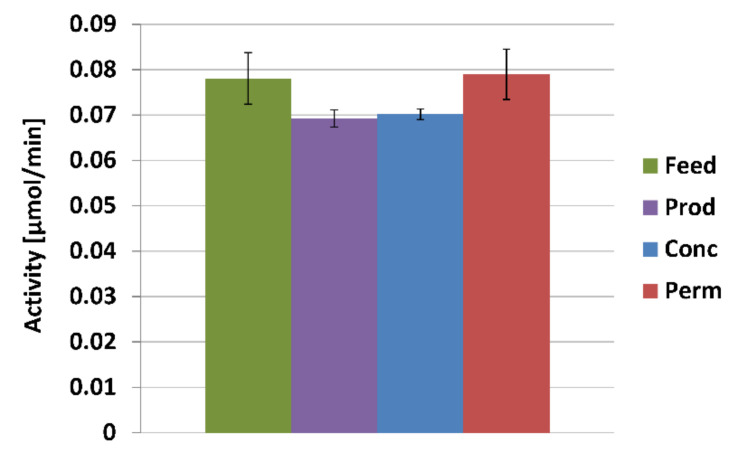
Oxygen consumption in aqueous media with lignosulfonate preparations and active laccase.

Reactions in aqueous solutions were monitored using an oxygen electrode (Figure 3). There was no significant difference (*t*-test, *p* ˂ 0.05) between the lignosulfonate fractions (Figure 3).

**Figure 4:**
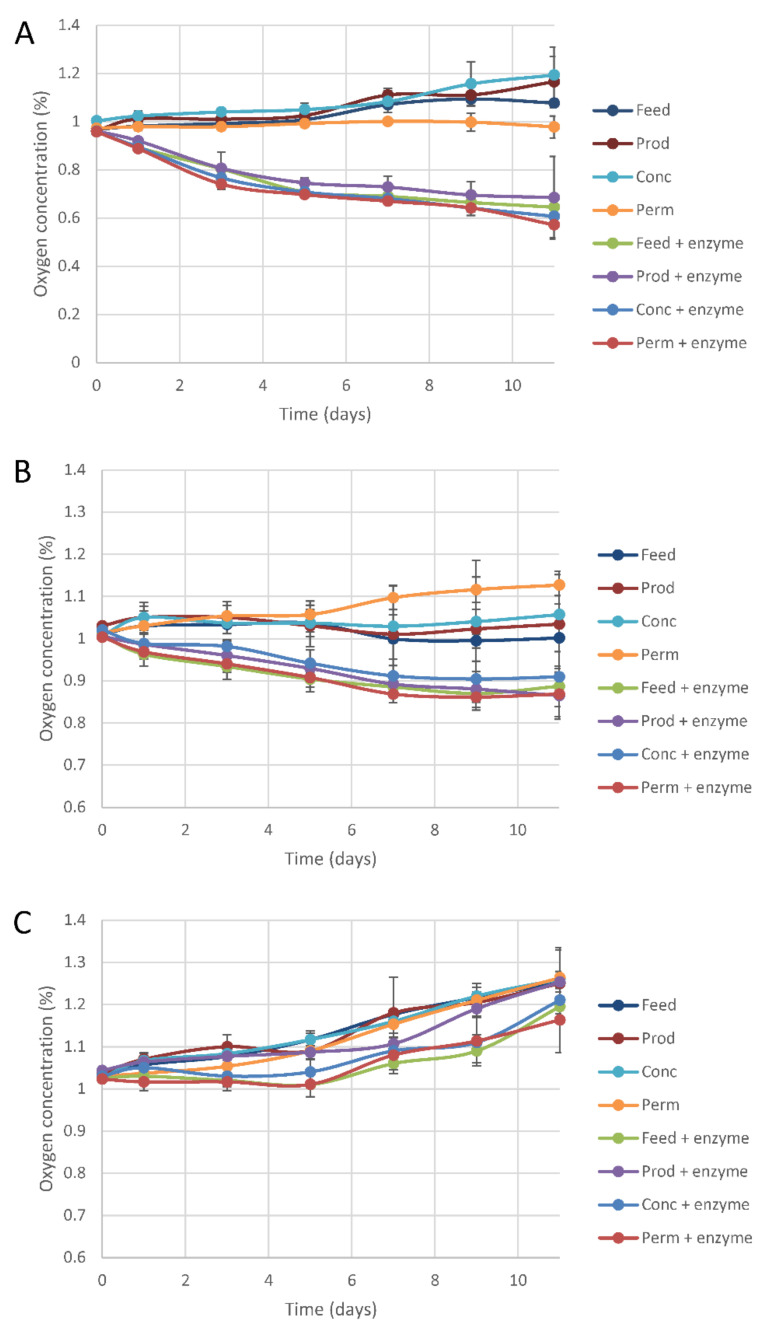
Oxygen contents of sealed reaction chambers containing coated boards with lignosulfonate preparations and active or denatured laccase during incubation at an RH of (**A**) 100%, (**B**) 92%, and (**C**) 84%.

Oxygen consumption by coated paperboards was monitored in sealed reaction chambers. The ability of the coatings to scavenge oxygen in the headspace of the sealed reaction chambers was evaluated at an RH (relative humidity) of 100%, 92%, and 84% (Figure 4, Table 4). Table 4 shows data after five days of incubation, as differences between reaction chambers with coatings with denatured and active laccase were fully or almost fully developed after that time period, and as standard deviations typically increased with time.

The results from the experiments performed at 100% RH are shown in Figure 4A and in Table 4. The differences between reaction chambers with active and denatured enzyme were very obvious (Figure 4A). After five days, the differences in oxygen content between reaction chambers with denatured and active enzyme were in the range 0.18–0.34% (Table 4, Series A). All these differences are statistically significant (*p* < 0.05). The results from the experiments performed at 92% RH are presented in Figure 4B and in Table 4. After five days, the differences in oxygen content between reaction chambers with denatured and active enzyme were in the range 0.10–0.15%. Although smaller than for 100% RH, the differences between reaction chambers with active and denatured enzyme were still obvious (Figure 4B) and, in most cases, statistically significant (*p* < 0.05) (Table 4, Series B). The results from the experiments performed at 84% RH are presented in Figure 4C and in Table 4. After five days, the differences in oxygen content between reaction chambers with denatured and active enzyme were in the range 0.07–0.11%, except for *Prod*, which did not exhibit any significant difference (Table 4, Series C). Thus, the experiments clearly showed differences between coatings with denatured and active enzyme and that the enzyme-catalyzed reaction waned with decreasing RH. The increase in oxygen that was apparent at the end of the experiments (Figure 4) suggests release of residual oxygen from the substrate or that there were some problems with leakage in the experiment. As reported values are mean values of triplicates, as experiments are based on comparisons between coatings with denatured and active laccase, and as most differences were statistically significant (Table 4), problems with residual oxygen or leakage should not have affected the conclusions drawn from the experiments.

The results on oxygen scavenging show that regardless of whether the reaction took place in liquid or solid medium (i.e., in buffered aqueous solution or in the film), the catalytic reaction in a given medium proceeded at a similar rate for all four lignosulfonate preparations. This can possibly be related to the similar phenolic contents of the four preparations, or to the supply of oxygen being a limiting factor.

The results show that oxygen scavenging using a system with laccase and fractionated lignosulfonates can be achieved at an RH of 92% and 100%. This system would therefore be suitable for packaging of foods with a water activity higher than 0.9 such as bread, cheese, fruit and meat [24]. In packages for fresh cut fruits and vegetables, an RH close to 100% is reached within a few hours when packed in polypropylene containers kept at a temperature of 5 °C [25].

**Figure 5:**
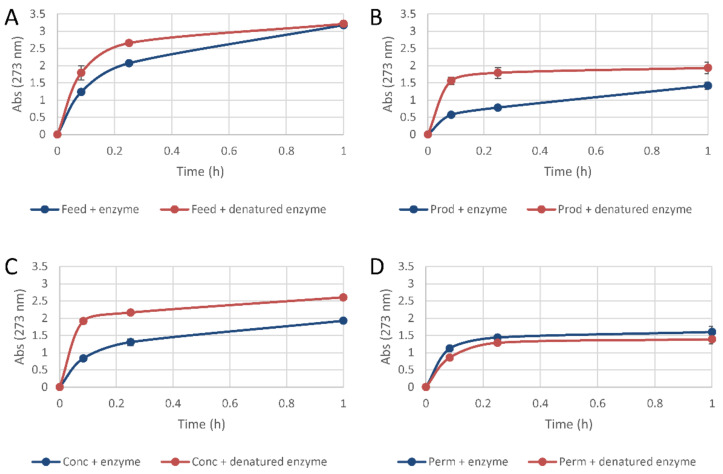
Water stability of films with lignosulfonate preparations [(**A**) *Feed*, (**B**) *Prod*, (**C**) *Conc*, (**D**) *Perm*] and active (blue symbols) or denatured (red symbols) laccase.

The results from the water stability test are shown in Figure 5. For *Prod* (Figure 5B) and *Conc* (Figure 5C), coatings containing active enzyme showed better water stability than coatings with denatured enzyme. Fractions rich in low-molecular-weight lignosulfonates, i.e., *Feed* (Figure 5A) and *Perm* (Figure 5D), did not show improved water stability. Studies of milled wood lignin from spruce have indicated that the fraction of phenylpropane units that contain phenolic hydroxyl group amounts to 0.24 [26]. Thus, far from all phenylpropane units in lignin contain phenolic hydroxyl groups that make them substrates for laccase-catalyzed reactions (without mediators). While larger lignin fragments would probably contain phenolic groups, small fragments would not necessarily contain phenolic groups and, therefore, would not necessarily serve as substrate for laccase.

## 3. Materials and Methods

### 3.1. Materials

Enzyme preparation: laccase from *Trametes versicolor* (*Tv*LCC) in powder form (Sigma-Aldrich, St Louis, MO, USA). Chemicals and polymers: 3-(*N*-morpholino)propanesulfonic acid (MOPS) (Sigma-Aldrich), pyrogallol (Sigma-Aldrich), SB-latex (styrene-butadiene latex) (Trinseo Europé GmbH, Horgen, Switzerland), clay (Barrisurf LX, Imerys Minerals Ltd., Cornwall, UK), lignosulfonates (kindly provided by Domsjö Fabriker AB, Örnsköldsvik, Sweden), starch (Solcoat P55 (hydroxypropylated and oxidized potato starch), Solam GmbH, Emlichheim, Germany), potassium chloride (Fluka, Buchs, Switzerland), potassium nitrate (Merck, Darmstadt, Germany), and glycerol ≥99.0% (Sigma-Aldrich). Board: Double-coated folding box board with uncoated reverse, grammage 255 g/m^2^ (Stora Enso, Helsinki, Finland).

### 3.2. Instruments

Spectrophotometer (UV-2101PC, Shimadzu, Kyoto, Japan), oxygen electrode (Oxygraph system, Hansatech Instruments, Kings Lynn, UK), bench coater K202 Control Coater (RK Print-Coat Instruments Ltd., Royston, UK), DMA/SDTA861 (Mettler Toledo, GmbH, Schwerzenbach, Switzerland), sealed reaction chambers (Mikrolab Aarhus A/S, Højbjerg, Denmark), CheckMate II (PBI Dansensor A/S, Ringsted, Denmark), freeze dryer (Heto Drywinner, Heto-Holten S/S, Allerød, Denmark), FTA 200 Dynamic Contact Angle Analyzer (First Ten Angstrom, Portsmouth, VA, USA), STFI thickness tester M201 (TJT Teknik AB, Järfälla, Sweden).

### 3.3. Fractionation of Lignosulfonates

The four industrially prepared lignosulfonate fractions that were used in the experiments were obtained from the Domsjö Fabriker AB wood biorefinery (Örnsköldsvik, Sweden) and are listed in Table 1. The biorefinery uses softwood (mainly Norway spruce) as feedstock for a sulfite process with sodium as base, and produces specialty cellulose for the textile industry, lignosulfonates, bioethanol, and other products. The lignosulfonate preparations used were: (1) *Feed*, crude lignosulfonates before fractionation with ultra filtration, (2) *Prod*, product fraction, enriched in lignosulfonates of intermediate molecular mass by ultra filtration (5–60 kDa) (retentate and permeate), (3) *Conc*, concentrated high molecular mass (≥60 kDa) fraction (retentate), and (4) *Perm*, permeate enriched in lignosulfonates of low-molecular mass (≤60 kDa). The fractions were freeze-dried and stored at room temperature.

### 3.4. Enzyme Activity Assay

The volumetric activity of the *Tv*LCC preparation was determined using the spectrophotometer and a colorimetric assay based on the oxidation of pyrogallol. One unit (U) was defined as the amount of enzyme needed for converting 1 µM of pyrogallol/min at 25 °C in an aqueous solution of 50 mM MOPS buffer (pH 6.5). The activity of *Tv*LCC in an aqueous solution with lignosulfonates as the substrate was determined using the oxygen electrode. The pH was 6.5 (50 mM MOPS) and the temperature was 25 °C.

### 3.5. Preparation of Paperboard Coatings

Latex-based coating colors were prepared as described by Johansson et al. [16] and contained the following components in pph (parts per hundred (by weight) of dry latex): 100 pph SB-latex, 55 pph clay, 30 pph lignosulfonates, 10 pph starch, and 1 pph *Tv*LCC. Coating colors with denatured enzyme were prepared as controls. The coating color was double-coated onto the top side (coated side) of the board using the bench coater and a wire-wound rod, giving a nominal wet-deposit of 24 µm. The drying procedure of the coating was 30 s at 105 °C in a ventilated oven.

### 3.6. Preparation of Starch-Based Films

Starch-based films were prepared essentially as described by Johansson et al. [16], but were modified as follows due to strong adhesion to the plastic in the petri dishes. The mixture contained the following components [in parts per hundred (by weight) of dry starch]: 100 pph starch, 55 pph clay, 20 pph glycerol, 20 pph lignosulfonate, and 1 pph enzyme preparation. Coating color with denatured enzyme was used as control and the enzyme was denatured by boiling for 12 min. The casting and the subsequent drying of films in the petri dishes was performed according to Johansson et al. [7].

### 3.7. Preparation of Powders

The reaction mixture contained 100 mg/mL lignosulfonate preparation (pH adjusted to 6.5), 2.5 U/mL laccase (*Tv*LCC), and 100 mM MOPS buffer (pH 6.5). For each reaction, seven mL of the reaction mixture were transferred to a 50-mL Falcon tube with pierced caps. The reaction mixtures were then incubated at 23 °C, 50% RH for 4, 8, 24, and 48 h with stirring. The reactions were terminated by freeze-drying. The 0 h sample was prepared by freeze-drying seven mL of the reaction mixture immediately after enzyme addition. Lignosulfonates with denatured enzyme and sampling after 0, 4, 8, 24, and 48 h were used as controls.

### 3.8. Analysis of Phenolic Content

The contents of phenolic groups were estimated using the method of Lai et al. [27]. The analysis was performed by MoRe Research AB (Örnsköldsvik, Sweden). The relative standard error was ≤10% and single samples were analyzed

### 3.9. GPC (Gel Permeation Chromatography)

The GPC analysis was performed by MoRe Research AB. The relative standard error was estimated at ≤10%. Single samples were analyzed. UV detection was carried out at 256 nm. The weight average molecular weight (M_w_) was estimated based on poly(styrenesulfonate) standards.

### 3.10. Contact Angle

The FTA 200 Dynamic Contact Angle Analyzer was used to determine the contact angle of 10 µL deionized water at 23 °C and 50% RH on cast films. The CCD camera was taking 40 snapshots during 80 s. The contact angle was determined on starch-based films and the measurement was performed in six replicates. The linear part was extrapolated to the y-intercept, and mean values and standard deviations were calculated. The cast films were conditioned at 23 °C and 50% RH overnight prior to analysis.

### 3.11. Mechanical Properties

The mechanical properties of cast starch-based films were evaluated using DMA (Dynamic Mechanical Analysis). The film was cut into pieces of 0.5 × 3 cm. The thickness was measured using the STFI thickness tester. An amplitude sweep was performed and an amplitude in the linear region was selected. The oscillation sweep was performed from 0.1 to 10 Hz in tension mode at a temperature of 23 °C and at an RH of 50%. The measurements were performed in six replicates. The cast films were conditioned at 23 °C and 50% RH overnight prior to analysis.

### 3.12. Activity in Sealed Reaction Chambers

The coated board was conditioned in air at 23 °C and 50% RH overnight (equilibrium was confirmed by two consecutive weighings of the board, carried out at an interval of time of >60 min) and cut into pieces of 21 × 6 cm. The pieces were pleated and placed into sealed reaction chambers. The atmosphere in the sealed reaction chambers with a volume of 110 mL was 1% oxygen and 99% nitrogen (AGA Gas AB, Enköping, Sweden). The oxygen-scavenging capacity of the coated boards was studied using a CheckMate II (zirconia sensor, the sample volume was 2 mL/sampling point) at different RH. The desired RH was created by placing inside the sealed reaction chamber a cup of 1.5 mL water (100% RH), or 1.5 mL saturated potassium nitrate (92% RH), or 1.5 mL saturated potassium chloride (84% RH). Coated boards without enzyme were used as control. The reactions were performed in triplicates and the mean value and standard deviation were calculated.

### 3.13. Water Stability

The water stability of the casted films was studied. The cast films were cut in pieces of 0.6 × 1 cm and placed in 15 mL Falcon tubes containing 5 mL of a solution of 50 mM MOPS (pH 6.5) and incubated at 23 °C with rotation at 22 rpm. The sampling was performed after 0, 5, 15, and 60 min, and the absorbance was measured in a spectrophotometer at 273 nm. The reactions were performed in triplicates and mean values and standard deviations were calculated.

## 4. Conclusions

The consequences of size fractionation of lignosulfonates prior to utilization as antioxidants in laccase-catalyzed oxygen-scavenging were investigated. Enrichment of lignosulfonates of larger size did not lead to decreased enzymatic activity, neither in aqueous solution nor in films. This is advantageous for the possibility to utilize lignosulfonates for active packaging, as the preparations enriched in high-molecular-weight components showed improved water stability and improved cross-linking giving a polymerization product with larger average molecular weight and increased hydrophobicity. Further research in this area may shed light on factors that affect the rate of the enzymatic reaction, and lead to new possibilities to use lignin derivatives for advanced high value-added products.

## Figures and Tables

**Table 1:** Lignosulfonate fractions.

Short Name	Enriched Fraction (kDa)	Brief Description
*Feed*	-	Crude, non-fractionated
*Prod*	5–60	Product fraction
*Conc*	≥60	Concentrated high Mw fraction
*Perm*	≤60	Low Mw fraction permeate

**Table 2:** The phenolic content of the lignosulfonate fractions.

Lignosulfonate Preparation	Phenolic Content % (*w*/*w*)
*Feed*	0.28
*Prod*	0.31
*Conc*	0.25
*Perm*	0.25

**Table 3:** The average molecular weight of the lignin derivatives in cast films with and without enzymatic treatment.

Sample	Mw	
	Active Enzyme	Denatured Enzyme
Film with *Feed*	23,400	6700
Film with *Prod*	66,000	5400
Film with *Conc*	31,600	6200
Film with *Perm*	17,600	3800

**Table 4:** Oxygen content (in %) and differences (Δ) in oxygen content between deactivated enzyme (DE) and active enzyme (AE) after five days at different relative humidity (RH) ^a^.

Series A	100% RH, DE	100% RH, AE	100% RH, ΔO_2_
*Feed*	1.01 ± 0.01	0.71 ± 0.02	0.30 *
*Prod*	0.99 ± 0.06	0.81 ± 0.01	0.18 *
*Conc*	1.05 ± 0.03	0.71 ± 0.01	0.34 *
*Perm*	0.98 ± 0.01	0.80 ± 0.01	0.18 *
**Series B**	**92% RH, DE**	**92% RH, AE**	**92% RH, ΔO_2_**
*Feed*	1.04 ± 0.02	0.90 ± 0.03	0.14 *
*Prod*	1.03 ± 0.01	0.93 ± 0.04	0.10
*Conc*	1.04 ± 0.03	0.94 ± 0.03	0.10 *
*Perm*	1.06 ± 0.05	0.91 ± 0.01	0.15 *
**Series C**	**84% RH, DE**	**84% RH, AE**	**84% RH, ΔO_2_**
*Feed*	1.12 ± 0.02	1.01 ± 0.01	0.11 *
*Prod*	1.09 ± 0.04	1.09 ± 0.02	ND
*Conc*	1.12 ± 0.02	1.05 ± 0.01	0.07 *
*Perm*	1.09 ± 0.02	1.01 ± 0.03	0.08 *

^a^ Asterisks (*) indicate significant differences (*p* < 0.05) with Student’s *t*-test. ND, not detected.

## Data Availability

Data is contained within the article.

## Sample Availability

Samples of the compounds are not available from the authors.
